# Multiple Abscess Collections: Antibiotics or Steroids?

**DOI:** 10.1155/2024/3671685

**Published:** 2024-01-25

**Authors:** Philippe Raphael Dias, Levin Bolt, Christof Iking-Konert, Mattia Arrigo, Lars C. Huber

**Affiliations:** ^1^Department of Internal Medicine, Stadtspital Zurich Triemli, Zurich, Switzerland; ^2^Division of Rheumatology, Stadtspital Zurich Triemli, Zurich, Switzerland

## Abstract

Aseptic abscess syndrome (AAS) is a medical rarity. The combination of multiple abscess collections in different organs, negative microbiological studies, and the association with an inflammatory bowel disease is highly suggestive for an AAS. The AAS is an acute neutrophilic dermatosis, so “generalized pyoderma gangraenosum” or “generalized bullous sweet syndrome” might be used synonymously. It is important to note that the diagnosis of an AAS can be made only after careful exclusion of an infectious disease. Of interest, despite the severity of the inflammation, patients with AAS are commonly hemodynamically stable. To date, no studies have investigated the optimal regimen, dose, and duration of therapy. Corticosteroids are the cornerstone of immunosuppression during the acute phase. After the induction phase, therapy might be switched to anakinra or infliximab.

## 1. Presentation

A 60-year-old man originating from Sri Lanka presented to our emergency department with upper abdominal pain and diarrhea, sometimes accompanied by small amounts of fresh blood. Personal history included hypothyroidism under substitution with levothyroxine and asthma treated with formoterol/budesonide as needed. Travel and environmental history were unremarkable, family history for inflammatory bowel disease (IBD) was negative, no allergies were reported, and the patient denied the use of nicotine, alcohol, or illicit drugs. Clinical examination was unremarkable, and routine laboratory analysis showed no signs of inflammation. Abdominal computed tomography displayed discrete thickening of the intestinal wall due to possible colitis. The patient was treated symptomatically, and a follow-up endoscopic investigation was planned in an outpatient setting. A few days before the endoscopy, the patient presented again with acute deterioration, fever, abdominal pain, and persistent watery and bloody diarrhea.

At readmission, the patient presented in markedly reduced general condition, subfebrile (37.9°C), hypotensive, and bradycardic (92/57 mmHg, 48 bpm). Respiratory rate and oxygen saturation while breathing ambient air were within normal ranges. Physical examination revealed a distended abdomen with normal bowel sounds and diffuse tenderness on palpation with defense. Cardiac and pulmonary auscultation were unremarkable.

**Figure 1:**
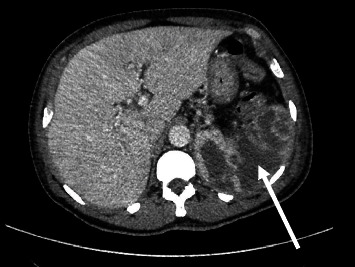
Splenic lesions (arrow).

**Figure 2:**
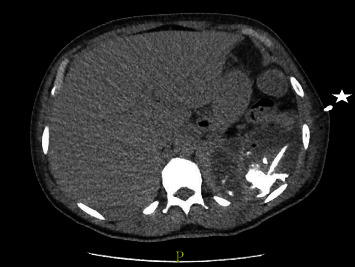
Splenic lesions after insertion of a drainage ( ^*∗*^).

Laboratory analysis now showed elevated leucocytes (10.7 × 10^9^/*µ*L) and C-reactive protein (225 mg/L), moderate normocytic normochromic anemia (hemoglobin 9.5 g/dL), elevated aminotransferases (ALAT 106U/L, ASAT 52U/L) and a mild hypokalemia. Serum lactate was normal. An abdominal CT scan showed progressive thickening of the intestinal wall. Additionally, splenic lesions, most likely of embolic origin, were found (Figures 1 and 2). Empiric antibiotic treatment with ceftriaxone and metronidazole was started, and a diagnostic test was performed.

## 2. Assessment

Recto-sigmoidoscopy revealed severe, left-sided erosive colitis. The biopsies showed moderate inflammation with neutrophilic crypt abscesses, indicating a diagnosis of ulcerative colitis. Oral and rectal mesalazine was added.

Transthoracic and transesophageal echocardiography were normal, with no evidence of endocarditis, intracardiac thrombus, or persistent foramen ovale.

Blood cultures showed no growth, and stool tests, including campylobacter, salmonella, shigella/EIEC, yersinia, clostridioides, parasites, and helminths, were negative. Serology for HIV, hepatitis A, B, C, EBV, CMV, and interferon-gamma tests were negative.

**Figure 3:**
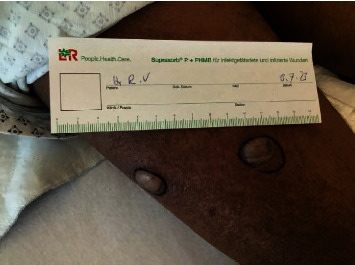
Multiple purulent skin lesions.

**Figure 4:**
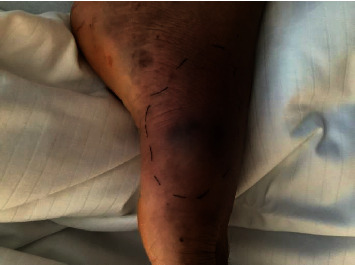
Metatarsal arthritis of the left foot.

In the following days, the general condition progressively deteriorated with persistent fever and increasing inflammatory markers. Repeated endoscopy showed improvement of the colitis, and a CT scan showed enlarging splenic lesions. Skin examination revealed purulent bullae at various locations (Figures 3 and 4). Of note, abscesses developed within a few hours at explicit sites following puncture (pathergy effect). In addition, pain, redness, and swelling of several joints (i.e., right knee, left elbow, left metatarsal, right hand) were observed. Arthrocentesis of the left elbow drained 15 mL of putrid fluid, and differential cell analysis showed 76,000 cells/*µ*L (95% neutrophils). A diagnostic puncture of the splenic lesions was performed and revealed a large amount of putrid fluid.

**Figure 5:**
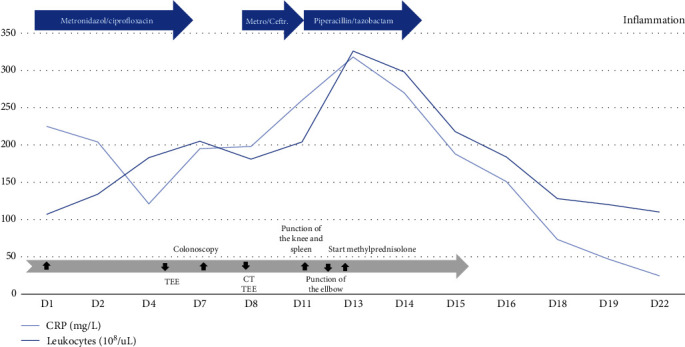
Timeline of inflammatory biomarkers, diagnostic investigations, and therapeutic regimens.

Empiric antibiotic therapy was escalated to piperacillin/tazobactam until negative microbiological test exams were received, including direct microscopy, culture, and eubacterial polymerase chain reaction. A timeline of inflammatory biomarkers, diagnostic investigations, and therapeutic regimens is provided in Figure 5. The patient remained hemodynamically stable.

## 3. Diagnosis and Management

The combination of multiple abscesses within different organs (spleen, joints, skin), negative microbiological evidence, and the association with an IBD was highly suggestive for the aseptic abscess syndrome (AAS), and a therapy with systemic corticosteroids was started.

In the following days, a rapid clinical improvement with prompt decrease of leukocytes and circulating C-reactive protein was observed (Figure 5). Intravenous methylprednisolone (62.5 mg q.d.) was switched to oral prednisone (50 mg q.d.) after 7 days with a slow tapering over the next weeks. Follow-up after 1 month showed an asymptomatic patient. Laboratory analysis showed normal leucocytes (9.9 × 10^9^/*µ*L) and C-reactive protein (3 mg/L). Hemoglobin and liver enzymes were normal. Ultrasound examination showed a residual splenic lesion of 2 cm and no signs of inflammation of the colon. Therapy was switched to the TNF-alpha inhibitor infliximab.

## 4. Discussion

AAS is a medical rarity. To our knowledge, about 30 case reports and a case series with 71 patients have been described to date [1]. In addition, Bollegala et al. [2] published a summary of patients with IBD-associated AAS from the literature databases.

The real case number, however, might be underestimated since “generalized bullous Sweet's syndrome” or “generalized pyoderma gangraenosum” are used synonymously. All these entities can be subsumed under the term of acute neutrophilic dermatosis [3]. The mechanisms of these disorders are not completely unraveled. Inadequate activation of neutrophilic granulocytes appears to be a final common pathway, resulting in the production of pus (cell detritus) at various localizations.

It is important to note that the diagnosis of an AAS can be made only after careful exclusion of an infectious disease. Moreover, AAS, generalized bullous Sweet syndrome, and generalized pyoderma gangraenosum as an extraintestinal manifestation of IBD are manifestations of a pathogenetic spectrum. As such, the distinction between these different entities is difficult and, in many cases, only of academic interest. Whole genome sequencing might be performed to confirm the diagnosis definitely. This, however, is not performed on a routine base due to a lack of direct clinical consequences and costs.

There are no studies that have investigated the optimal regimen, dose, and duration of therapy. Most patients are highly inflamed but hemodynamically stable. Empirical antibiotic therapy is commonly initiated, which can be stopped after the exclusion of an infectious genesis. Corticosteroids (e.g., prednisone 1 mg/kg/day) are the cornerstone of immunosuppression during the acute phase of the disease. After the induction phase, therapy might be switched to cyclophosphamide, azathioprine, anakinra, or infliximab [4]. In the lack of clinical data, we recommend the use of anakinra because of the auto-inflammatory nature of AAS or infliximab due to the excellent effects observed in chronic inflammatory bowel disease and pyoderma gangraenosum [5, 6]. Furthermore, both drugs have a fast mode of action and a well-known side effect profile. Anecdotal reports have described prolonged remission after splenectomy [7].

## References

[1] Trefond L., Frances C., Costedoat-Chalumeau N. (2022). Aseptic abscess syndrome: clinical characteristics, associated diseases, and up to 30 years’ evolution data on a 71-patient series. *Journal of Clinical Medicine*.

[2] Bollegala N., Khan R., Scaffidi M. A. (2017). Aseptic abscesses and inflammatory bowel disease: two cases and review of literature. *Canadian Journal of Gastroenterology and Hepatology*.

[3] Wallach D. (2005). Les dermatoses neutrophiliques neutrophilic dermatoses. *La Revue de Médecine Interne*.

[4] Elessa D., Thietart S., Corpechot C., Fain O., Mekinian A. (2019). TNF-*α* antagonist infliximab for aseptic abscess syndrome. *La Presse Médicale*.

[5] Antonelli E., Bassotti G., Tramontana M. (2021). Dermatological manifestations in inflammatory bowel diseases. *Journal of Clinical Medicine*.

[6] Wollina U., Haroske G. (2011). Pyoderma gangraenosum. *Current Opinion in Rheumatology*.

[7] Doll R., Friedman K., Hostoffer R. (2018). Aseptic abscess syndrome, a case of prolonged remission following splenectomy. *American Journal of Gastroenterology*.

